# From low sense of control to problematic smartphone use severity during Covid-19 outbreak: The mediating role of fear of missing out and the moderating role of repetitive negative thinking

**DOI:** 10.1371/journal.pone.0261023

**Published:** 2021-12-22

**Authors:** Julia Brailovskaia, Jan Stirnberg, Dmitri Rozgonjuk, Jürgen Margraf, Jon D. Elhai

**Affiliations:** 1 Mental Health Research and Treatment Center, Department of Clinical Psychology and Psychotherapy, Ruhr-Universität Bochum, Bochum, Germany; 2 Department of Molecular Psychology, Ulm University, Ulm, Germany; 3 Institute of Mathematics and Statistics, University of Tartu, Tartu, Estonia; 4 Department of Psychology, University of Toledo, Toledo, Ohio, United States of America; 5 Department of Psychiatry, University of Toledo, Toledo, Ohio, United States of America; Aalborg University, DENMARK

## Abstract

Since the outbreak of Covid-19, the use of digital devices, especially smartphones, remarkably increased. Smartphone use belongs to one’s daily routine, but can negatively impact physical and mental health, performance, and relationships if used excessively. The present study aimed to investigate potential correlates of problematic smartphone use (PSU) severity and the mechanisms underlying its development. Data of 516 smartphone users from Germany (*M*_*age*_ = 31.91, *SD*_*age*_ = 12.96) were assessed via online surveys in April and May 2021. PSU severity was significantly negatively associated with sense of control. In contrast, it was significantly positively linked to fear of missing out (FoMO), repetitive negative thinking (RNT), and daily time spent on smartphone use. In a moderated mediation analysis, the negative relationship between sense of control and PSU severity was significantly mediated by FoMO. RNT significantly moderated the positive association between FoMO and PSU severity. Specifically, the higher the RNT, the stronger the relationship between FoMO and PSU. The present findings disclose potential mechanisms that could contribute to PSU. Potential ways of how to reduce PSU severity are discussed.

## Introduction

During the 21^st^ century, smartphones became people’s daily companions. Through mobile Internet access, smartphones allow permanent availability and provide up-to-date news around the globe and in the life of family and friends [1]. Oral communication through phone calls is complemented by typed interaction and exchange of photos and videos anywhere and at any time using various social media applications on our smartphone—that is social platforms such as Facebook, Instagram, and Twitter, as well as instant messengers such as WhatsApp, Telegram, and Signal [2]. In addition to the active online interaction, one can passively observe the online behavior of others by checking their updates. Depending on privacy settings, we can track when our friends are online, and for example whether they have read our recent messages on WhatsApp [3, 4]. To sum up, through the smartphone we can participate in the lives of other people and allow them to be part of our lives [5]. These functions of our smartphone not only satisfy our need for belonging; they also can contribute to the satisfaction of another important human need—the sense of control [6, 7].

Sense of control is an essential element of humans [8]. People want to control the course of events in the own lives and to decide on their own what to do, where to go, with whom to meet and how to present themselves [9, 10]. The lack of control over important life events can be experienced as a high psychological burden and foster symptoms of anxiety, depression, and helplessness [11–13]. Individuals who lack functional coping strategies to regain control, or to manage life despite loss of control, often resort to inadequate and dysfunctional strategies such as substance abuse, overeating or restrictive eating. In the short-term, these strategies seem to reduce negative emotions and provide some control; but in the longer-term, they negatively impact mental and physical health and contribute to interpersonal problems [14–16]. Nowadays, people who experience loss of control in important life areas often gravitate to using digital devices (that is, often and over a long period of time), especially smartphones, as a form of coping strategy [e.g., 17, 18].

Smartphone use allows individuals to escape negative feelings, to forget overwhelming problems of everyday life at least temporarily, and to experience positive emotions [19]. The permanent possibility of social interaction and observation of others’ online behavior can reduce the individual perception of control loss [7, 20, 21]. Previous research assumed that the positive effects can be maintained as long as the intensity of smartphone use remains moderate. However, its increase may negatively impact the individual health and behavior [22].

The Interaction of Person-Affect-Cognition-Execution (I-PACE) model for addictive behavior provides a theoretical framework for this assumption [23]. Following this model and further available research, in the longer-term, due to the interaction between various mediating and moderating factors, excessive use can contribute to development of a strong emotional bond to the digital device, closely associated with habit formation of prolonged smartphone use [21, 23–25]. If this happens, the individual may tend to progressively engage in excessive smartphone use in different situations as an impulsive response, even if alternative behavior would be more functional, reasonable and productive [23]. In addition, non-use can provoke negative consequences such as mental and physical withdrawal, mood deterioration, aggressive behavior, and an unconscious grasp of the smartphone [26, 27].

This phenomenon has been termed as compulsive, dependent, addictive, or problematic smartphone use (PSU) [28]. It is defined by characteristics such as salience, tolerance, mood modification, relapse, withdrawal, and interpersonal conflicts [2, 24]. The characteristics are close to substance abuse [29]. However, addictive use of smartphones has not been recognized as a formal psychiatric disorder in the Diagnostic and Statistical Manual of Mental Disorders (DSM-5; [30]) or International Classification of Diseases (ICD-11; [31]), so far. Moreover, earlier research warned of over-pathologization of the impact of intensive media use on mental health [32, 33]. Against this background and considering the current lack of a standardized term, we will use the term PSU as proposed by Panova and Carbonell [34] and avoid terms such as “addictive smartphone use” in the current study.

Available cross-sectional and longitudinal studies have detailed negative mental and physical health of PSU severity, as well as adverse effects on daily performance in different areas [35, 36]. For instance, PSU severity is positively correlated with depression, anxiety, irritability, and suicide ideation, and it may reduce happiness and life satisfaction [for overview, see 37]. Time spent on intensive smartphone use was negatively associated with sleep quality and sleepiness during bedtime. Its link to daytime sleepiness was positive [7, 36, 38]. Screen time during bedtime was positively related to PSU severity [39]. Furthermore, time spent on smartphone use was negatively related to individual fitness and physical activity, and positively to obesity [40–42]. In addition to enhanced interpersonal problems [43], PSU severity was positively linked to increased procrastination, and reduced academic and work performance [44–46].

Considering these findings and the high involvement of smartphones in everyday life, it seems highly desirable and relevant to investigate potential correlates of PSU severity and the mechanisms linked to its development (mediation and moderation effects) and, therefore, to be able to identify and to protect people at risk for this form of problematic behavior.

In the present study, we will specifically focus on the relationship between sense of control due to important events in everyday life and PSU, because both—sense of control and smartphone use—have become of specific relevance during the global outbreak of the coronavirus disease (Covid-19; severe acute respiratory syndrome coronavirus 2, SARS-CoV-2) in the year 2020 [47, 48].

Notably, the outbreak and rapid spread of Covid-19 required significant changes in everyday life worldwide [49]. Since the beginning of 2020, restrictive rules to fight the pandemic were introduced by many governments and authorities around the globe. Many of those restrictions remained applicable in the course of 2021 [50, 51]. The rules included closing public institutions, recreational venues, shops, and non-essential businesses, bans on traveling and non-family gatherings, overnight or fulltime curfews, as well as behavioral measures such as maintaining distance from other people (“social distance”), and wearing of face masks [52, 53]. While some people quickly adapted to the required changes, others experienced them as a significant psychological burden and loss of control of their daily routine [54, 55].

The need for “social distance” and enhanced staying at home due to home-working and home-schooling resulted in enhanced use of digital devices, especially smartphones, to obtain news, stay in touch with others outside the home via telephony and social media, and as recreation by playing games and watching videos [47, 56]. Also the characteristics of problematic use increased [57]. Based on earlier research [7, 58], it can be assumed that, on the one hand, people who experience the Covid-19 crisis as a significant loss of control might engage in PSU as a dysfunctional coping strategy. On the other hand, individuals with high sense of control might be less at risk for PSU. They could experience the Covid-19 pandemic as less burdensome and tend to functional coping strategies (e.g., maintaining daily routine as much as possible, enhancement of physical activity) [59].

The negative link between sense of control and PSU severity could be impacted by different factors. Based on previous research (e.g., [60, 61]), one such factor might be the fear of missing out (FoMO) that is defined as “pervasive apprehension that others might be having rewarding experiences from which one is absent” ([62]; p. 1841). FoMO is closely associated with the strong desire to know what other people—especially friends—are doing. Satisfaction of this desire contributes to the individual sense of control. The lack of such information and connection can evoke stress, frustration, and uneasiness and negatively impact mental health [25, 63–65]. High levels of FoMO can result in dysfunctional coping strategies such as alcohol abuse [66, 67]. Smartphones that connect us with social media via mobile Internet allow us to permanently update our knowledge of others’ lives, and thus to stay socially involved. Notably, this technical development also means that updates might be missed when we do not visit the online world. This, however, can contribute to enhanced FoMO [62, 68]. As a consequence, FoMO can be reduced by a permanent checking of other people’s updates via the smartphone which, however, may foster the characteristics of PSU such as salience, tolerance and withdrawal [69].

The Covid-19 crisis requires “social distance” to slow down the pandemic spread. Possibilities of social interaction and of receiving updates from other people in-person are strongly reduced. This might enhance the FoMO that results in permanent checking of online updates via smartphone, and, thus, at least partly explain the increase of PSU [18]. Following available findings, low sense of control is positively linked to the experience of FoMO [67]. Furthermore, recent research showed that individuals who experience the Covid-19 crisis and required changes in daily routine as a significant control loss are prone to problematic media use [6]. Therefore, we hypothesized that individuals with low sense of control could be at enhanced risk for FoMO, and thus also for PSU. Specifically, FoMO could mediate the relationship between sense of control and PSU.

A further factor that might be involved in the relationship between sense of control and PSU is repetitive negative thinking (RNT). RNT describes the tendency of perseverative thinking about negative experiences and problems that is partly intrusive and difficult to disengage from [70]. Rumination and worry are two forms of RNT [71]. Rumination is defined as a repetitive thinking in response to sadness and depressiveness. The person focuses on the meaning and implications of the negative mood, and the past events that caused this mood [72]. In contrast, worry refers to negative affect-laden, mostly uncontrollable repetitive thinking that focuses on events with the potential for future negative outcomes [73]. RNT is closely linked to anxiety and depression [71, 74]. It belongs to predictors of dysfunctional coping strategies in stressful situations, for example of problematic alcohol consumption [75, 76]. Moreover, earlier research found a positive association between RNT and PSU severity [77, 78]. Recent studies showed that especially individuals who tend to worry and rumination experience the uncertainly and unpredictability of the Covid-19 crisis as a high burden [59, 79]. By trying to cope with the negative emotions, some of them tend to problematic smartphone use [80]. Furthermore, a positive relationship between rumination and FoMO was reported [81, 82].

Considering the definitions of FoMO [62] and RNT [83], we conceptualized that the negative effect of FoMO on mental health and behavior might be enhanced in persons who tend to RNT. This assumption can be explained as follows. The fear of missing out on important experiences (including important news) is the definition characteristic of FoMO [84]. This fear might also be accompanied by negative mood and thoughts. For instance, because of the missed experience, one could lose connection to other people—the person might no longer belong to the in-group connected by a mutual experience. In addition, one might lose important future opportunities [85]. This could foster specific activities to reduce the aversive state such as PSU that belongs to the easiest ways to do so during the Covid-19 crisis [18]. In individuals with high levels of RNT who are prone to sad mood and an uncontrollable repetition of negative thoughts [71], the urge for these activities—that in the short-term seem to reduce the aversive state—could be especially high. In contrast, people who are less prone to RNT could tend to less dysfunctional coping strategies even if they experience FoMO. Against this background, we hypothesize that RNT could moderate the positive association between FoMO and problematic smartphone use. Specifically, the higher the RNT, the closer the link between both variables.

Considering the presented background and our aim to investigate the mechanisms that are linked to PSU, we formulated the following hypotheses:

Sense of control is expected to be negatively associated with FoMO (Hypothesis 1a) and PSU severity (Hypothesis 1b). FoMO is assumed to be positively related to PSU severity (Hypothesis 1c).
Furthermore, FoMO is expected to mediate the relationship between sense of control and PSU severity (Hypothesis 2).
RNT is assumed to be positively associated with FoMO (Hypothesis 3a) and PSU severity (Hypothesis 3b).
Moreover, RNT is assumed to moderate the relationship between FoMO and PSU severity (Hypothesis 4). That is, the higher the level of RNT, the closer the positive link between FoMO and PSU severity.

**Fig 1:**
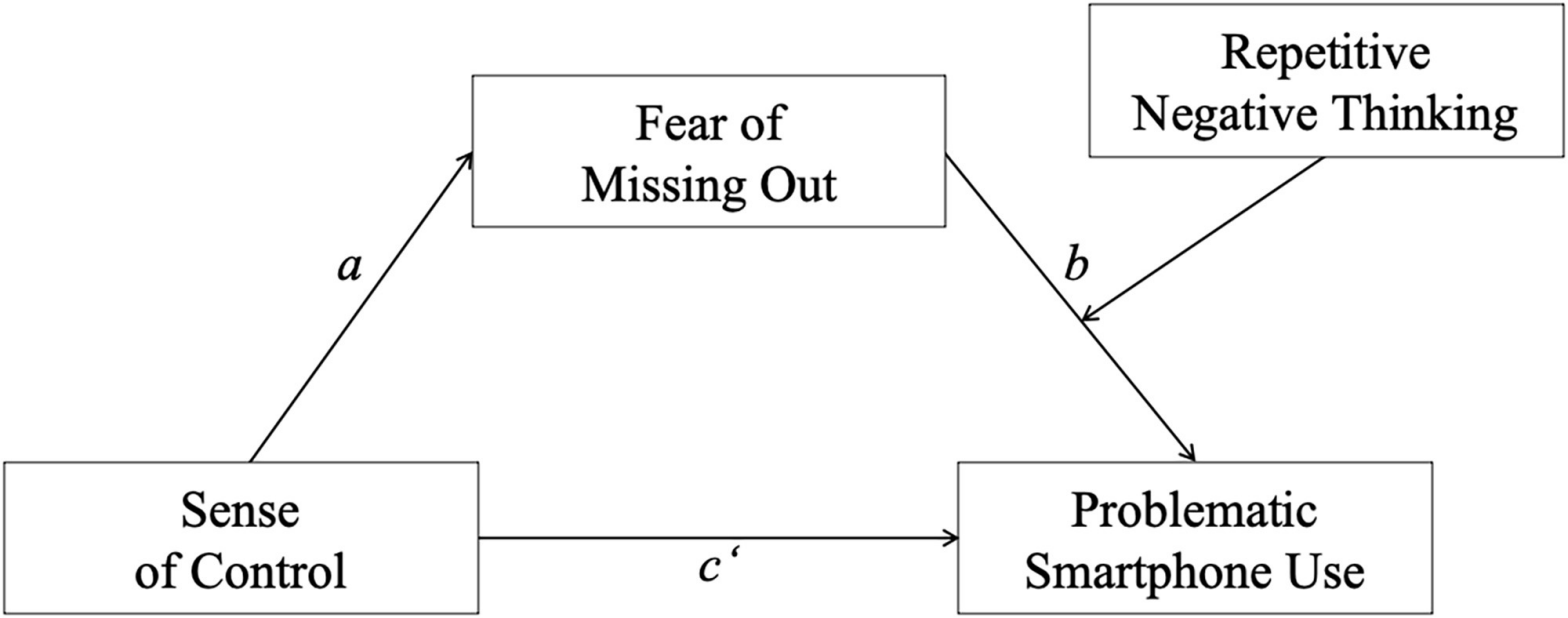
Moderated mediation model with sense of control (predictor), fear of missing out (mediator), repetitive negative thinking (moderator) and problematic smartphone use (outcome); age, gender, and daily smartphone use time (covariates).

Fig 1 illustrates the hypothesized relationships as a moderated mediation model (cf., [86]; p. 450).

We investigated the hypotheses in a sample from Germany. In spring 2021, there were about 3.5 million Covid-19 cases in Germany [87]. The first national Covid-19 lockdown was introduced in March 2020 in this country. The timeline and extent of the lockdown differed between the federal German states. In some states curfews were imposed, while other states only encouraged a “stay-at-home” and home-working requirement. Nationalwide, public gatherings of more than two non-family members were prohibited, mass events were cancelled, many public establishments and services were closed, school and university teaching was transferred to online classes (home-schooling). The wearing of face masks in public places and the keeping of 1.5m (4.9 ft) distance to non-family members became compulsory [88, 89]. After an easing of some measures in summer and autumn 2020, they became effective again with the beginning of the second national Covid-19 lockdown that was introduced in December 2020 in all German states. A slow easing of the measures began only in the end of spring 2021 [90, 91].

## Materials and methods

### Procedure and participants

The current sample comprised 516 smartphone users from Germany (75.2% women; *M*_age_ = 31.92, *SD*_age_ = 12.96, range: 18–79; occupation: 48.6% students, 3.9% apprentices, 45% employed, 1% unemployed, 1.6% retired). Data were collected between April and May 2021 via an online survey in German language. Participants were recruited by participation invitations displayed at several universities in Germany, on social media (Facebook, Twitter), and at public places (like bakeries, shops). Participation was voluntary, and required ownership of a smartphone and legal age due to German law (i.e., age of 18). University students were compensated by course points. All participants were provided instructions and gave informed consent to participate via an online form. The study implementation was approved by the Ethics Committee of the Faculty of Psychology of the Ruhr-Universität Bochum. The survey was completed by 516 (97%) of the 532 people who started it. No data were excluded. The dataset used in the present study is available in S1 Dataset.

### Measures

#### Sense of control

Following Niemeyer, Bieda [92] sense of control was assessed with the original German language items “Do you experience important areas of your life (i.e., work, free-time, family, etc.) to be uncontrollable, meaning that you cannot, or barely can, influence them?” and “Do you experience these important areas of your life as unpredictable or inscrutable?”. The two items are rated on a 5-point Likert-type scale (0 = *not at all*, 4 = *very strong*). For the calculation of the sum score, both items were reversed. Higher sum scores indicate higher sense of control. Earlier reported scale reliability was Cronbach’s *α* = .820 [48]; the scale reliability in the present study was *α* = .792.

#### Fear of Missing Out (FoMO)

The Fear of Missing Out Scale (FoMO Scale; original version: [62]; German language version: [93]) assessed FoMO with ten items (e.g., “I get anxious when I don’t know what my friends are up to”). The items are rated on a 5-point Likert-type scale (1 = *not at all true of me*, 5 = *extremely true of me*). Higher sum scores indicate higher FoMO. Earlier reported scale reliability was *α* = .770 [93]; the scale reliability in the present study was *α* = .831.

#### Repetitive negative thinking

Following Brailovskaia, Margraf [94] the level of RNT was assessed with two original German language items that were construed based on available longer RNT measures (Perseverative Thinking Questionnaire, PTQ; [70]). The items focused, respectively, on one of the two RNT forms—worry (“I am often worried”) and rumination (“I often tend to ruminate”). The items are rated on a 5-point Likert-type scale (1 = *does not apply to me at all*, 5 = *applies to me very much*). The higher the sum score, the higher the level of RNT. Notably, previous research emphasized the validity, reliability, and efficiency of single-item instruments and encouraged their use [95–97]. Earlier reported scale reliability was *α* = .830 [94]. The scale reliability in the present study was: *α* = .851.

#### Problematic smartphone use

PSU was assessed with a modified version of the brief Bergen Social Media Addiction Scale (BSMAS; original version: [98]; German version: [99]). Earlier research reported the BSMAS to have good psychometric properties (scale reliability: *α* = .880 [100]). In the six items that are formulated according to the six characteristics of problematic social media use (i.e., salience, tolerance, mood modification, relapse, withdrawal, conflict) the term “Social Media” was replaced by “Smartphone” (e.g., “Felt an urge to use the Smartphone more and more?”). The items are rated on a 5-point Likert-type scale (1 = *very rarely*, 5 = *very often*). Higher sum scores indicate higher problematic smartphone use. The scale reliability in the present samples was *α* = .843.

Furthermore, participants were asked to rate how much time they daily spent on smartphone use (in minutes) (i.e., “How much time do you on daily spent on smartphone use?”).

### Statistical analyses

Statistical analyses were conducted using SPSS 26 and the Process macro version 3.5 (www.processmacro.org/index.html). After descriptive analyses, associations between the investigated variables were assessed by zero-order bivariate correlations. Next, a moderated mediation analysis that included a conditional indirect effect (see Fig 1) was computed (Process: model 14). This analysis examined the multiple effects simultaneously (integration of the hypothesized mediation and moderation models) [101, 102]. The moderated mediation effect was assessed by the bootstrapping procedure (10,000 samples) that provides percentile bootstrap confidence intervals (*CI* 95%). The analysis included sense of control as predictor, FoMO as mediator, RNT as moderator, and PSU as outcome (see Fig 1). Younger age, female gender and time spent on smartphone use were previously reported to be positively related to PSU [37]. Thus, considering the rather young and female composition of our sample, we controlled for age and gender by including both as covariates in the moderated mediation model. In addition, we also controlled for daily smartphone use time. Path *a* denoted the link between sense of control and FoMO; the relationship between FoMO and PSU was denoted by path *b*; path *c’* (the direct effect) denoted the association between sense of control and PSU after the inclusion of FoMO and RNT in the model.

## Results

Table 1 presents descriptive statistics of the investigated variables and their correlations. The correlation analyses revealed that sense of control was significantly negatively correlated with FoMO, RNT, and PSU severity (all: *p* < .001), as well as with time spent daily on smartphone use (*p* < .05). Furthermore, there were significant positive correlations between FoMO, RNT, smartphone use time and PSU severity (all: *p* < .001) (see Table 1).

**Table 1:** Descriptive statistics and correlations of sense of control, fear of missing out, repetitive negative thinking, smartphone use time, and problematic smartphone use.

	*M (SD)*	*Min–Max*	Skewness	Kurtosis	(2)	(3)	(4)	(5)
(1) Sense of Control	5.34 (1.83)	0–8	-.474	-.234	-.295**	-.377**	-.105*	-.234**
(2) Fear of Missing Out	22.60 (7.12)	10–49	.532	.151		.359**	.326**	.527**
(3) Repetitive Negative Thinking	6.29 (2.15)	2–10	-.007	-.742			.230**	.358**
(4) Smartphone Use Time	193.54 (94.86)	6–480	.492	.048				.432**
(5) Problematic Smartphone Use	13.04 (5.19)	6–30	.610	-.238				

*N* = 516; Smartphone Use Time = daily smartphone use time in minutes; *M* = Mean; *SD* = Standard Deviation; *Min* = Minimum, *Max* = Maximum (both are empirical values of the present study);

**p < .001,

*p < .05.

**Fig 2:**
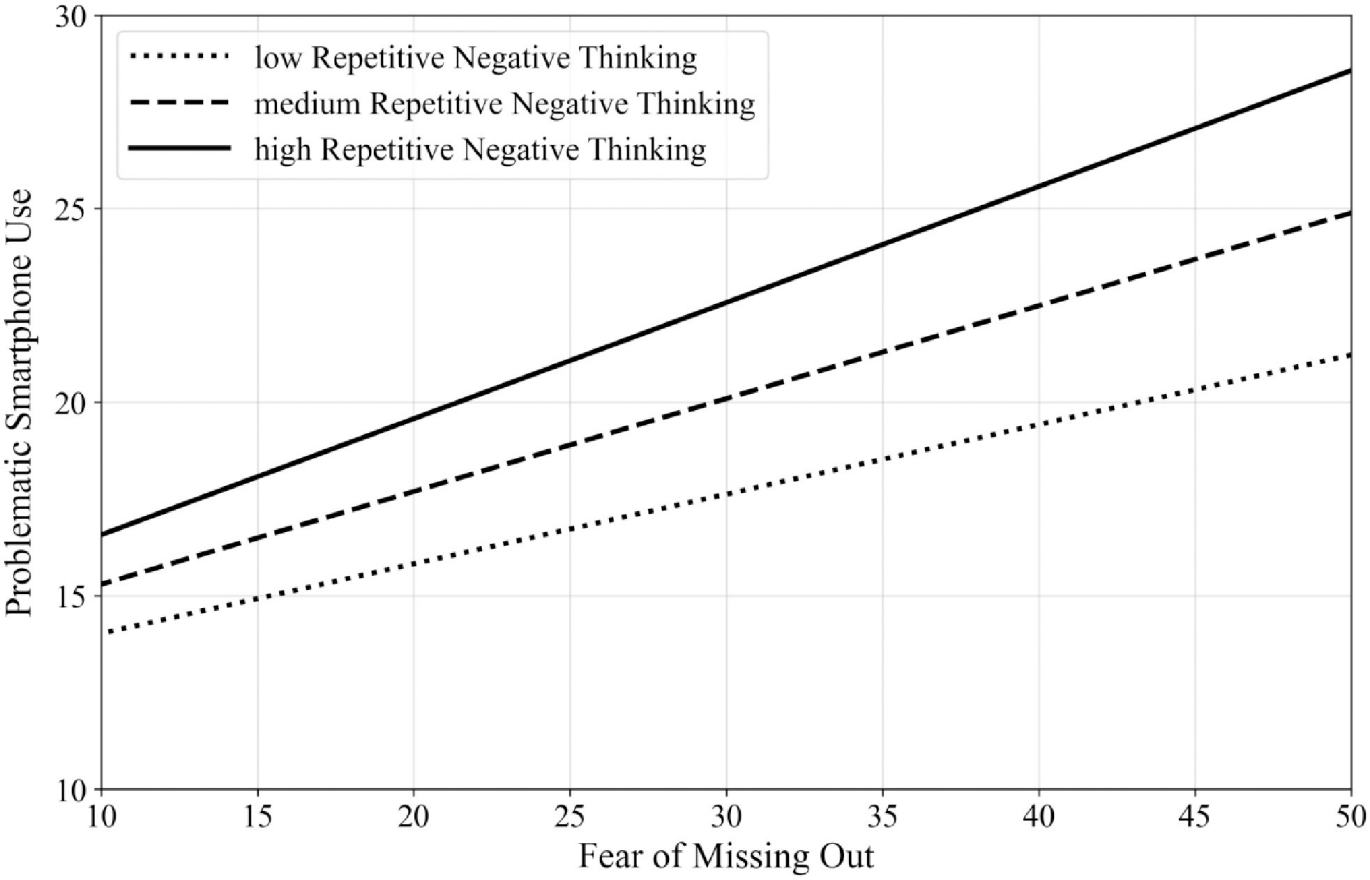
Moderating effect of repetitive negative thinking on the connection between fear of missing out and problematic smartphone use (*N* = 516).

The moderated mediation analysis showed significant findings (see Table 2). The overall model was significant, *F*(5,508) = 54.433, *p* < .001, *R*^*2*^ = .391. The direct effect (path *c’*) of sense of control on PSU severity was not significant (*p* = .250) after controlling for FoMO, RNT, and their interaction, as well as the covariates age, gender and daily time spent on smartphone use. The conditional indirect effect of sense of control on PSU severity through FoMO was significant in participants with low, medium, and high levels of RNT. However, as shown in Table 2, this effect was stronger for participants with high rather than medium or low levels of RNT (effect: high level > medium level > low level). Fig 2 visualizes the moderation effect of RNT on the link between FoMO and PSU severity.

**Table 2:** Moderated mediation model (outcome: Problematic smartphone use).

	ß	SE	t	*p*	95% *CI*
*Model 1*: *Outcome Problematic Smartphone Use*					
Path *a*: Sense of Control → FoMO	-.968	.167	-5.813	< .001	[-1.295, -.641]
Path *b*: FoMO → Problematic Smartphone Use	.240	.031	7.763	< .001	[.179, .301]
Interaction: FoMO*RNT → Problematic Smartphone Use	.026	.012	2.191	.029	[.003, .049]
Path *c’* (direct effect): Sense of Control → Problematic Smartphone Use	-.137	.119	-1.151	.250	[-.371, .097]
*Conditional Indirect Effects*: *Sense of Control* → *Problematic Smartphone Use*					
Sense of Control → FoMO → Problematic Smartphone Use					
RNT:					
Low (one SD below mean = -2.148)	-.178	.049			[-.280, -.089]
Medium (mean = 0)	-.232	.049			[-.335, -.141]
High (one SD above mean = 2.148)	-.286	.062			[-.416, -.172]
*Index of Moderated Mediation*	-.025	.012			[-.051, -.002]

*N* = 516; covariates: age, gender, daily smartphone use time (in minutes); RNT = Repetitive Negative Thinking; FoMO = Fear of Missing out; SD = Standard Deviation; ß = Standardized Beta, SE = Standard Error, t = t-test, *p* = significance, *CI* = Confidence Interval.

## Discussion

Smartphone use can facilitate one’s daily routine and reduce loneliness, but it can also have negative impact on health, work, and relationships [37]. As shown by recent research, during the Covid-19 outbreak the role of smartphones on life remarkably increased, as well increased the problematic characteristics of smartphone use [18, 56, 57]. Against this background, the current study investigated potential correlates of PSU severity during the Covid-19 outbreak and the mechanisms that might explain PSU to contribute to the identification of individuals at risk and to protect them. Our findings reveal that sense of control, FoMO and repetitive negative thinking are significantly associated with PSU severity. Furthermore, they show how the investigated variables could interact.

As expected, sense of control was negatively associated with FoMO (confirmation of Hypothesis 1a) and PSU severity (confirmation of Hypothesis 1b). FoMO was positively related to PSU severity (confirmation of Hypothesis 1c). Moreover, FoMO served as a mediator between sense of control and PSU severity (confirmation of Hypothesis 2). Notably, our cross-sectional study design does not reveal causal conclusions. But based on available research [25, 67, 103, 104], our results allow the hypothetical assumption that individuals who experience loss of control of important life events could be at risk for enhanced levels of FoMO. To reduce this negative emotional state, they could consequently engage with their smartphones. The gratification experienced by this behavior could contribute to habit formation of prolonged smartphone use. In the longer-term, this might foster impulsive use and the development of PSU [23]. In contrast, people with high sense of control could be less prone to FoMO and PSU. Considering the negative association between sense of control and time spent daily on smartphone use in the present study, they could spend more time on adaptive activities that are not linked to smartphone use and be less preoccupied with issues that happen online. Thus, those without substantial negative affect, and with perceived self-control should have enough fulfilling and satisfying experiences that they would not feel compelled to experience FoMO or PSU to alleviate distress. This assumption is supported by previous research that described individuals who regularly engage in fulfilling leisure activities to have low levels of FoMO and problematic Internet use [105]. Moreover, satisfying leisure activities can contribute to one’s sense of control [106].

Thus, it might be that people, who engage in meaningful (leisure) activities during the required stay-at-home period since the Covid-19 outbreak, experience the overall situation as less burdensome and keep an adequate level of sense of control. Therefore, they experience less FoMO and engage less in PSU. Notably, sports engagement belongs to leisure activities that foster sense of control, physical and mental health, and can reduce problematic media use [107–109]. In a recent cross-national study, physical activity reduced the negative impact of depression symptoms on the experience of psychological burden by the Covid-19 crisis [59]. Thus, regular physical activity that does not require expensive equipment (e.g., jogging, gymnastics, yoga) could be an adequate way to protect us against PSU.

Furthermore, we found a positive association between RNT and FoMO (confirmation of Hypothesis 3a), as well as between RNT and PSU severity (confirmation of Hypothesis 3b). Moreover, RNT served as a moderator between FoMO and PSU (confirmation of Hypothesis 4): The higher the RNT, the closer the positive link between FoMO and PSU severity. The present results allow the hypothetical assumption—due to the cross-sectional data no causal conclusion can be drawn—that worry and rumination as subtypes of RNT [71] could foster the negative mood and thoughts involved with FoMO, and therefore contribute to PSU. Thus, people who typically engage in RNT could be at enhanced risk for PSU and its potential negative consequences. In contrast, smartphone use of individuals who are less prone to RNT might be less problematic, even if they experience FoMO.

RNT is a transdiagnostic construct that is common for different mental disorders [74]. Moreover, previous research described that RNT can amplify the effect of risk factors on psychopathological outcomes, for example the development of depression [110]. Our findings correspond to available studies that reported RNT to be also positively linked to FoMO and PSU severity [77, 78, 80–82]. Furthermore, they complement this knowledge by showing for the first time that RNT could serve as a moderator between FoMO and PSU severity. Thus, we can assume that factors that reduce RNT could also contribute to the protection against PSU.

Mindfulness is described as the enhanced attention to and nonjudgment awareness of the current moment [111]. It belongs to the factors that can reduce RNT and is often included in psychotherapeutic treatment [112, 113]. Earlier research showed that mindfulness can contribute to the reduction of the negative impact of intensive social media use on mental health and on work performance [79, 114], and it can also reduce problematic social media use [115] and PSU severity [116]. Against this background, mindfulness might be a further protective factor against PSU. Its training and cultivation in everyday life [117] could be specifically beneficial for people with low sense of control, high FoMO and the tendency to RNT.

The present study has some limitations that need to be considered when interpreting the results. First, the cross-sectional online survey design does not allow true conclusions on causality; only hypothetical assumptions are possible. Therefore, our findings should be extended by longitudinal/experimental investigations. For example, in could be investigated by an experimental study design, whether the reduction of participants’ level of RNT by mindfulness practice [112] can influence the association between FoMO and PSU. Second, our sample was assessed only in Germany, and participants were mostly female and rather young. This might bias the results and limit their generalizability to other populations. Age and gender were controlled for in the moderated mediation analysis which partly tackled this limitation. Nevertheless, future studies are suggested to replicate our investigation in more age and gender balanced samples from other countries. Third, we assessed data via self-report that can be prone to social desirability and perception mistakes. The inclusion of a social desirability measure (for example, Balanced Inventory of Desirable Responding (BIDR; [118]) and additional measure of objective data (for example, usage of applications that assess the daily time spent on smartphone use) could tackle this limitation in future studies. Fourth, available research described that the Covid-19 crisis including restrictive governmental measures to slow down the pandemic spread significantly changed the everyday routine of many people in Germany (e.g., [119]) as well as in other countries (e.g., [47, 120]). Most of our participants were students or employees. In spring 2021, university lectures in Germany were held online and many employees were advised for home-working [90, 91]. However, we did not explicitly assess whether the daily routine of our participants in the present study changed during the Covid-19 crisis. Thus, we can only speculate whether they experienced the Covid-19 crisis and required changes in daily routine as a significant control loss.

To conclude, the present study reveals that low sense of control might contribute to PSU. Enhanced experience of FoMO combined with increased levels of RNT could reinforce this association. Thus, activities—for instance physical and mindfulness exercises (both can be practiced despite Covid-19-related restrictions on everyday life)–that allow positive experiences in the offline world and thus contribute to the increase of sense of control, reduction of RNT and FoMO might foster less PSU. This is of specific importance during the Covid-19 outbreak that has been accompanied by enhanced smartphone use in many countries.

## Supporting information

S1 Dataset Dataset used for analyses in present study. (SAV)
